# 

**Published:** 1892-07-23

**Authors:** 


					i'CE
fflV.E TWOPENCE.
vol. xii.-
WT6? at tv.
-July 23rd, 1892.
?werai roet umc0 fti a newspaper ror
11 m the United Kingdom and Abroad.]
UIH ELAIKH IMUK&lNU jSUrrueiVieiNI<
Per Annum, 8s. 6d., post free.
Monthly Farts, 6d.; post free, 8d.
Ditto per Annum, 8s.. post free.

				

